# Current insight on the mechanisms of programmed cell death in sepsis-induced myocardial dysfunction

**DOI:** 10.3389/fcell.2023.1309719

**Published:** 2023-12-15

**Authors:** An-Bu Liu, Shu-Jing Li, Yuan-Yuan Yu, Jun-Fei Zhang, Lei Ma

**Affiliations:** ^1^ Department of Emergency Medical, General Hospital of Ningxia Medical University, Yinchuan, Ningxia, China; ^2^ Department of Pediatrics Medical, General Hospital of Ningxia Medical University, Yinchuan, Ningxia, China

**Keywords:** sepsis induced myocardial injury (SIMI), programmed cell death (PCD), multi-target treatment, complication, infection

## Abstract

Sepsis is a clinical syndrome characterized by a dysregulated host response to infection, leading to life-threatening organ dysfunction. It is a high-fatality condition associated with a complex interplay of immune and inflammatory responses that can cause severe harm to vital organs. Sepsis-induced myocardial injury (SIMI), as a severe complication of sepsis, significantly affects the prognosis of septic patients and shortens their survival time. For the sake of better administrating hospitalized patients with sepsis, it is necessary to understand the specific mechanisms of SIMI. To date, multiple studies have shown that programmed cell death (PCD) may play an essential role in myocardial injury in sepsis, offering new strategies and insights for the therapeutic aspects of SIMI. This review aims to elucidate the role of cardiomyocyte’s programmed death in the pathophysiological mechanisms of SIMI, with a particular focus on the classical pathways, key molecules, and signaling transduction of PCD. It will explore the role of the cross-interaction between different patterns of PCD in SIMI, providing a new theoretical basis for multi-target treatments for SIMI.

## 1 Introduction

Sepsis is a clinical syndrome characterized by a dysregulated host response to infection, resulting in life-threatening organ dysfunction. It is a significant global health concern and poses a serious threat to human health worldwide (Wang et al., 2020a). Previous studies have indicated that the annual incidence of adult sepsis in 27 developed countries was approximately 288 cases per 100,000 population. Additionally, the annual incidence of severe sepsis is estimated to be around 270 cases per 100,000 population, with a case fatality rate of approximately 26% (Fleischmann et al., 2016). Previous analyses have demonstrated that the incidence and mortality of sepsis are higher in developing and less developed countries (Markwart et al., 2020), particularly among vulnerable groups such as pregnant women, infants, the elderly, and immunodeficient individuals (Lin et al., 2018; Fleischmann et al., 2021). This is often attributed to their relatively weaker immune function and limited access to healthcare resources. Indeed, there is a scarcity of current surveillance statistics on sepsis in low- and middle-income countries. Further research and surveillance efforts are crucial to address this knowledge gap and improve sepsis management worldwide. For decades, the mortality rate of sepsis has been declining with the development of science and technology. The sepsis patients’ mortality in intensive care units (ICUs) of developed countries has decreased from 35.0% in 2000 to 18.4.6% in 2012 (Kaukonen et al., 2014). Despite advancements in the understanding and management of sepsis, early diagnosis and treatment remain challenging due to the complex nature of the condition, which involves multiple etiologies and can result in damage to multiple organs. Therefore, it is imperative to investigate the mechanisms and pathophysiology underlying sepsis, as this forms the fundamental basis for enhancing the prognosis of septic patients.

The high mortality rate associated with sepsis is strongly correlated with the occurrence of several severe complications, including septic shock, multiple organ dysfunction syndrome (MODS), severe sepsis, and sepsis-induced myocardial injury (SIMI). These complications significantly contribute to the worsening of patient outcomes and pose significant challenges in the management of sepsis (Court et al., 2002; Levy, 2007). When septic shock occurs, the heart, as a vital component of the circulatory system, is one of the primary organs affected.

Parker and Parker et al., 1984 observed that 50% of patients with sepsis exhibited a reduction in the initial left ventricular ejection fraction (EF), accompanied by an increase in end-systolic and end-diastolic volumes. As a result, they proposed the concept of sepsis-induced myocardial injury (SIMI) The clinical manifestations of SIMI lack specificity, with common presentations including left ventricular dilation caused by infection, decreased left ventricular ejection fraction (LVEF), hemodynamic instability, and rapid cardiac arrhythmias, insufficient blood pressure elevation with adequate fluid resuscitation, poor response to catecholamine drugs, and inadequate organ tissue perfusion. However, due to the incomplete understanding of the pathogenesis of SIMI, there is currently a lack of evidence-based therapeutic measures. Instead, treatment primarily focuses on addressing the underlying diseases based on clinical best practices. Currently, the primary treatment strategies for SIMI may include fluid resuscitation, vasopressors, beta-blockers, anti-inflammatory therapy, and extracorporeal membrane oxygenation (ECMO). However, the effectiveness of these treatments and the prognosis of SIMI patients are often unsatisfactory. It has been observed that septic patients with cardiac dysfunction have a remarkably high mortality rate of up to 70%, whereas the mortality rate for septic patients without cardiac dysfunction is only 20% (Rabuel and Mebazaa, 2006). SIMI can be also recognized as one of the detrimental consequences of sepsis. In recent years, researchers have conducted clinical and fundamental studies on SIMI. These studies aim to enhance our understanding of the underlying mechanisms and pathophysiology of SIMI, in order to develop effective diagnostic methods and therapeutic strategies for managing this specific complication of sepsis.

In a 2020 study on cardiac cells, it has been found that atrial tissue contains 30.1% cardiomyocytes, 24.3% fibroblasts, 17.1% pericytes and smooth muscle cells, 12.2% endothelial cells and 10.4% immune cells. In comparison, the ventricular region includes 49.2% ventricular cardiomyocytes, 21.2% mural cells, 15.5% fibroblasts, 7.8% endothelial cells, and 5.3% immune cells (Litviňuková et al., 2020). Therefore, we can conclude that cardiomyocytes constitute the highest proportion in both the atria and ventricles. Previous study has revealed that the major cardiac pathological changes during sepsis may include myocardial infiltration by immune cells, subendocardial hemorrhage, interstitial and intracellular edema, endothelial cell edema, microcirculatory fibrin deposition, as well as focal myofibrillar dissolution, cardiomyocyte necrosis and interstitial fibrosis (Lv and Wang, 2016). Additionally, the accumulation of lipids within the cytoplasm of cardiomyocytes has also been observed in infective cardiomyopathy (Lv and Wang, 2016). Hence, most current studies on the molecular and underlying mechanisms of SIMI have focused on cardiomyocytes. To date, some researches have revealed that myocardial suppression, sympathetic nervous system activation, mitochondrial damage, and calcium homeostasis imbalance all contribute to the development of SIMI (Yang and Zhang, 2021; Rudiger et al., 2013; Zanotti-Cavazzoni and Hollenberg, 2009; Lin et al., 2020) Bi et al., 2022 has elucidated that the pathogenesis of SIMI mainly includes apoptosis, mitochondrial damage, autophagy, excessive inflammatory response, oxidative stress and pyroptosis. Moreover, Kakihana et al., 2016 have identified two main aspects of the mechanism underlying SIMI. On the one hand, there is a downregulation of β-adrenergic receptors caused by cytokines, nitric oxide, and other substances. This downregulation inhibits the signaling pathways that occur after receptor activation, resulting in a weakened adrenergic response at the level of cardiomyocytes. On the other hand, SIMI can be triggered by programmed or non-programmed cell death induced by various factors including toxins, complement activation, damage-associated molecular patterns (DAMPs), and certain myocardial inhibitory factors.

**FIGURE 1:**
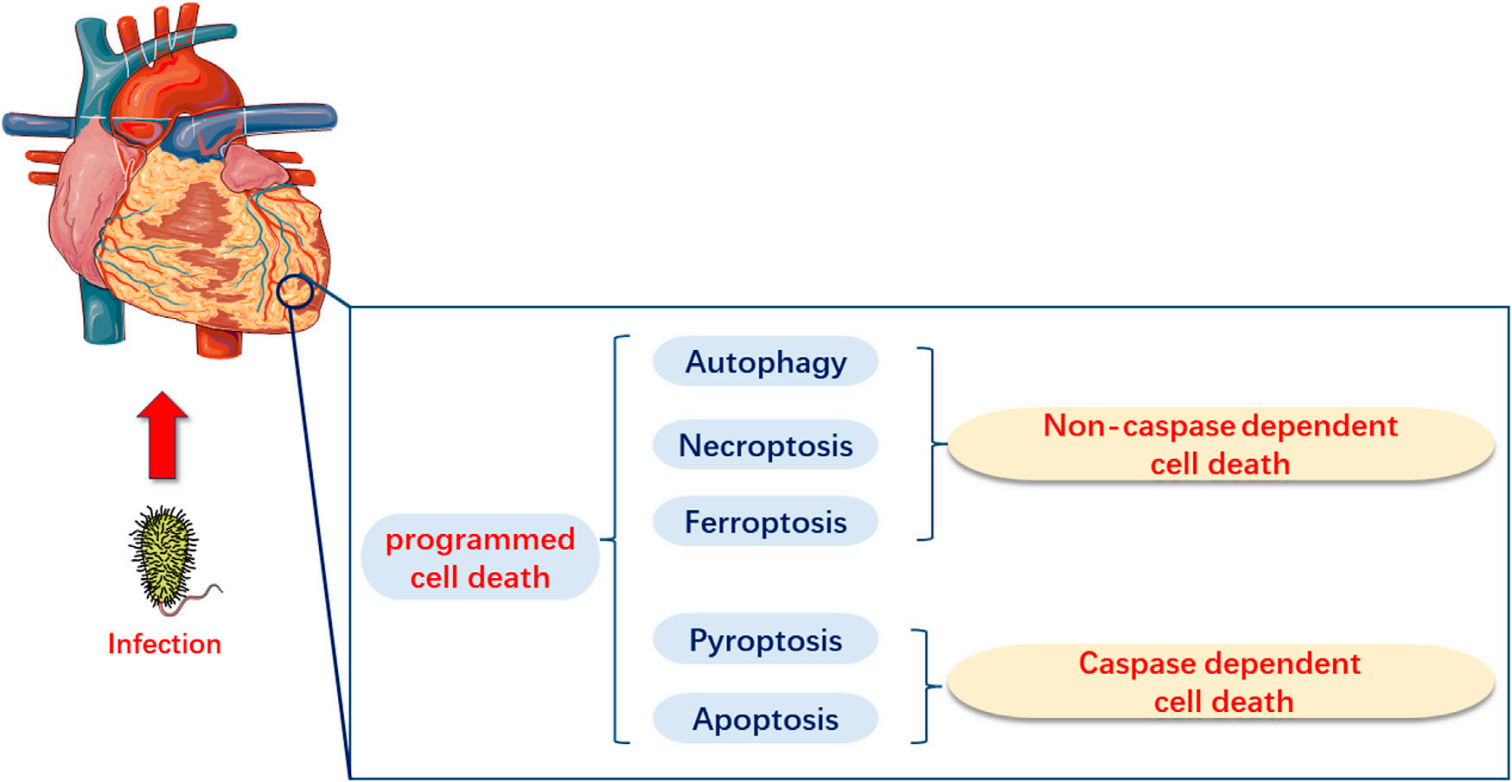
Programmed Cell Death in Myocardial Injury Induced by Sepsis (SIMI). In SIMI, programmed cell death can be categorized into caspase-dependent cell death, which includes apoptosis and pyroptosis, and non-caspase-dependent cell death, which includes ferroptosis and autophagy.

PCD, as a type of cell death, is mainly categorized into caspase-dependent cell death, such as apoptosis and pyroptosis, and non-caspase-dependent cell death, including necroptosis, ferroptosis, and autophagy (Tang et al., 2019a; Kopeina and Zhivotovsky, 2022). Several basic studies have demonstrated that apoptosis, necroptosis, pyroptosis, ferroptosis, and autophagy may have significant implications in the pathogenesis of sepsis and SIMI (Figure 1). However, current research directions have been limited to elucidate the role of one or several types of PCD in the pathogenesis of SIMI, which is relatively fragmented. The review not only comprehensively elaborated the role of the five types of PCD in SIMI from the perspective of signal transmission and key molecules, but also emphasized that these five types of PCD are interconnected rather than independent, collectively contributing to the occurrence and progression of SIMI. Furthermore, based on the interactions between PCDs, the review indicates that multi-target therapy may be a promising treatment strategy for SIMI in the future.

## 2 Apoptosis in SIMI

Apoptosis is one of the major characteristics of immune system dysfunction and a prominent feature in human sepsis and experimental cellular and animal models of sepsis (Ward, 2008). Therefore, apoptosis can be considered as one of the molecular mechanisms underlying SIMI, which is currently a widely discussed topic (Fernandes et al., 2008). It has been demonstrated in septic animal models that targeted interventions aimed at apoptosis significantly prolong the survival time of rats (Aziz et al., 2014). Based on the aforementioned content, we will present a comprehensive and focused description of apoptosis in the context of SIMI specifically highlighting apoptotic pathways, classical apoptotic signaling pathways and apoptotic proteins.

### 2.1 Pathways of apoptosis in SIMI

**FIGURE 2:**
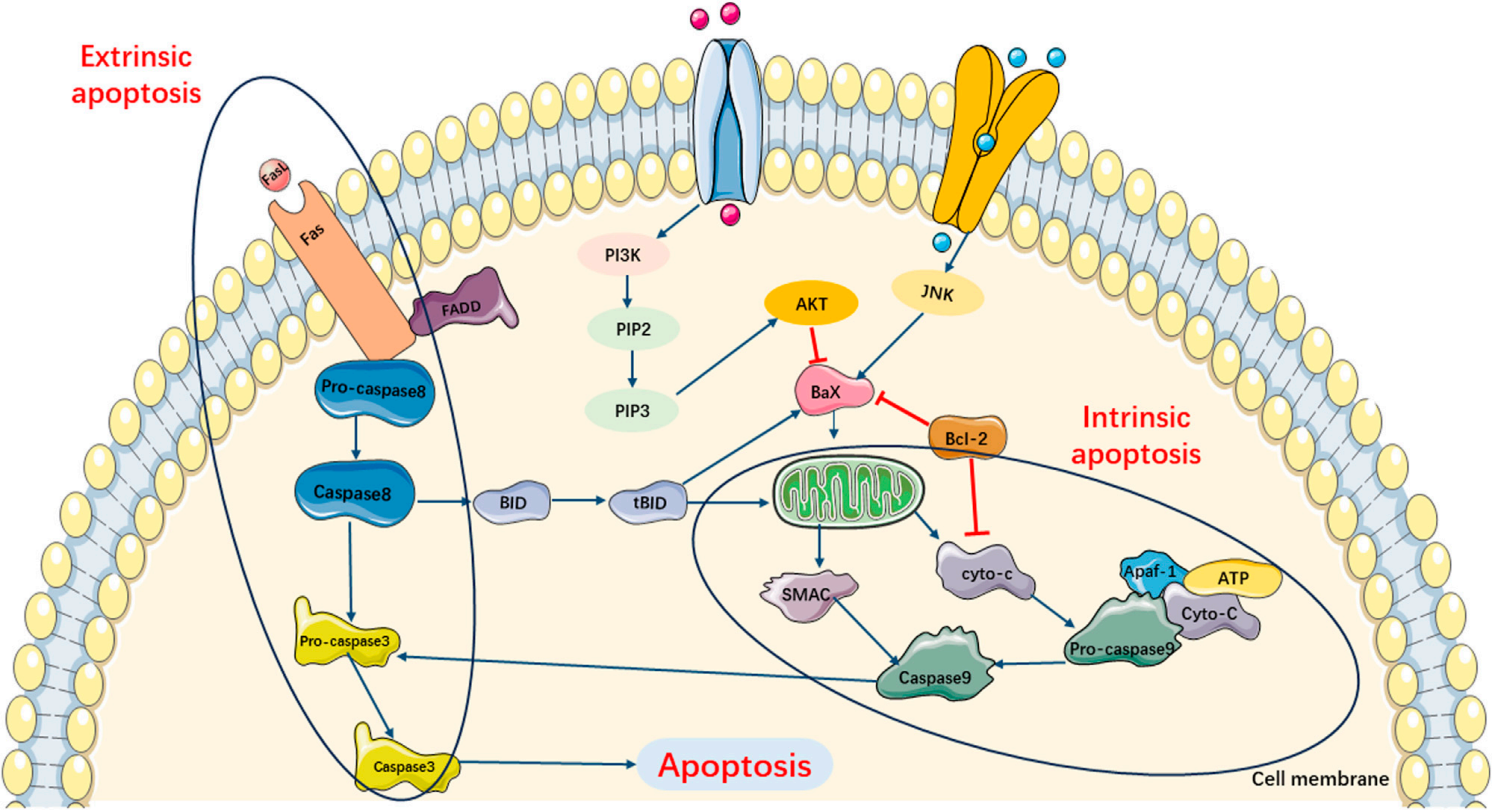
Main Pathways of Apoptosis. Pathway of apoptosis can be divided in extrinsic and intrinsic apoptotic cascade pathway. Extrinsic apoptotic cascade pathway is primarily activated through the specific activation TRADD receptors and caspase-8. Intrinsic apoptotic cascade pathway can be triggered by the release of cytochrome c and SMAC, which subsequently leads to increasing expression of pro-apoptotic protein Bax and induces cell apoptosis. Moreover, the combination of released cyto-c with Apaf-1 and caspase-9 also triggers exogenous apoptotic cascade reactions.

Previous studies have shown that there are two main apoptotic pathways involved in the development of SIMI (Levy and Deutschman, 2004; Bratton et al., 2000). The first pathway, also known as the extrinsic apoptotic cascade pathway, is primarily activated through the specific activation of tumor necrosis factor (TNF) receptor-associated death domain (TRADD) receptors and caspase-8. The second apoptotic pathway is primarily activated by stress-inducing stimuli. When the organism is subjected to various stresses, abnormal secretion of certain biochemical substances and cytokines occurs. This induces impaired mitochondrial function, resulting in the release of cytochrome C and Smac, which subsequently leads to an increase in the expression of the pro-apoptotic protein Bax and induces cell apoptosis. Moreover, the combination of released cytochrome C with Apaf-1 and caspase-9 triggers endogenous and exogenous apoptotic cascade reactions (Figure 2).

### 2.2 Classical signaling pathways of apoptosis in SIMI

**FIGURE 3:**
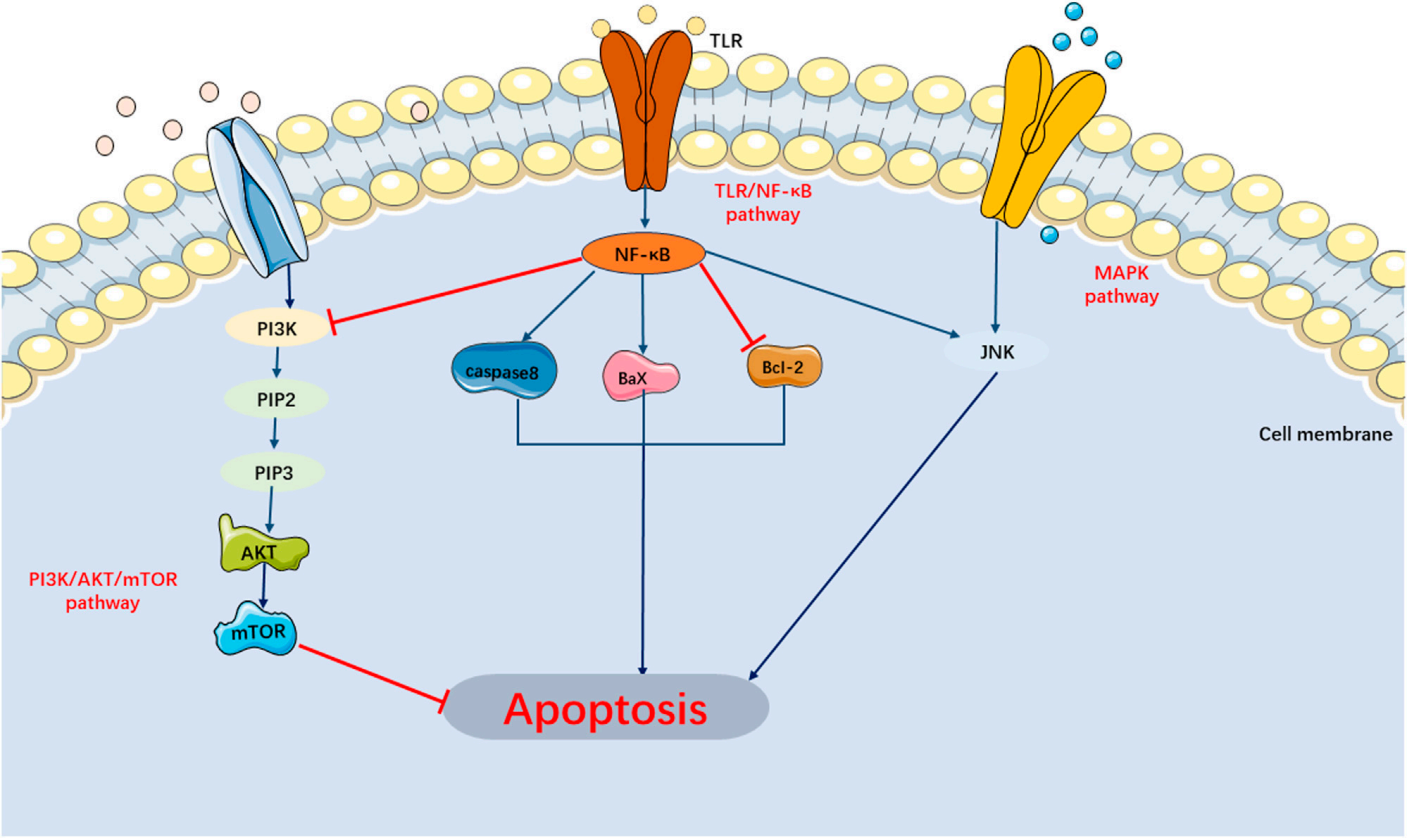
Main Signaling Pathways of Apoptosis. MAPK and TLR/NF-κB pathway can activate apoptosis. PI3K/AKT/mTOR pathway can inhibit apoptosis.

Currently, researches on the apoptosis signaling pathways in SIMI have primarily focused on the MAPK, PI3K/AKT/mTOR, and TLR/NF-κB signaling pathways (Figure 3).

### 2.3 MAPK pathway

Studies have demonstrated that mammalian cells have the ability to recognize and respond to extracellular stimuli by activating mitogen-activated protein kinases (MAPKs) through specific signaling cascades, eliciting specific cellular responses. MAPKs have been implicated in a variety of cellular processes, including mitosis, cell survival, apoptosis, cell differentiation, and cell proliferation. Studies have revealed that MAPK activation contributes to enhanced production of TNF-α and apoptosis in a model of LPS-induced cardiomyocytes (Liu et al., 2009). As of now, five members of the MAPK family have been identified. Among them, ERK1/2, JNK, and p38-MAPK are the most extensively researched members. Studies have demonstrated the involvement of JNK and p38-MAPK in the enhancement of TNF-α production and apoptosis in cardiomyocytes induced by endotoxins. Apoptosis signal-regulating kinase 1 (ASK-1) activated JNK and p38-MAPK through mitochondria-dependent caspase-3, leading to apoptosis. This process involved the upregulation of phosphorylated anti-apoptotic protein Bcl-2 and the release of cytochromeC. Suppression JNK/Bax signaling pathway can mitigate SIMI (Yang et al., 2014a). Furthermore, it has been discovered that activation of the TNF-α/P38-MAPK/calpain I signaling pathway can induce cardiomyocyte apoptosis in SIMI (Martin and Ranieri, 2011a; Zhang et al., 2016a). In addition, activating transcription factor 2 (ATF-2), a member of the ATF/cAMP response element-binding protein family, is a pro-apoptotic transcription factor. It became phosphorylated in the presence of p38-MAPK and JNK, leading to the translocation of NF-κB from the cytoplasm to the nucleus (Livingstone et al., 1995). Growing evidence suggested that the activation of NF-κB and MAPK contributed to an enhanced production of TNF-α and increased apoptosis in cardiomyocytes when exposed to LPS (Wang et al., 2015). Typically, the transcription factor NF-κB remained inactive as it bound to the repressor protein I-κB located in the cytoplasm. However, upon activation by LPS, NF-κB translocated to the nucleus, subsequently inducing the transcription of various genes associated with inflammation and apoptosis (Pham et al., 2004). Previous evidence suggested that in LPS-induced myocardial injury mice, miR-101-3p was upregulated and suppressed its specific target, Dual Specificity Phosphatase-1 (DUSP1), thereby further activating the MAPK and NF-κB signaling pathways and inducing apoptosis (Xin et al., 2021).

However, the role of ERK1/2, a member of the MAPK family, appears to be somewhat controversial. Previous studies have indicated that ERK has an opposite role compared to JNK and p38 MAPK (Yu et al., 2014). Similarly, Li et al., 2019a demonstrated that in the LPS-induced SIMI model, phosphorylation of JNK and p38 MAPK was increased, while phosphorylation and expression of ERK were slightly decreased. However, other studies have revealed that ERK, JNK, and p38 MAPK were all activated through phosphorylation in LPS-induced septic cardiomyopathy (Shao et al., 2021). Therefore, further investigations are warranted to elucidate the precise role of ERK1/2 in the apoptotic mechanism underlying septic myocardial injury.

### 2.4 PI3K/AKT/mTOR pathway

The phosphatidylinositol 3-kinase (PI3K)/protein kinase B (AKT) pathway can be activated by external stimuli, resulting in the phosphorylation of downstream signaling molecule AKT, which in turn phosphorylates mammalian target of rapamycin (mTOR), a downstream signaling molecule. This mechanism may play a critical role in regulating various physiological and pathological processes in the body, including inflammation, cell proliferation, autophagy, and apoptosis (Wang et al., 2019a; Franke et al., 1997; Jafari et al., 2019). Previous studies have indicated that activation of the PI3K signaling pathway can suppress cardiomyocyte apoptosis and mitigate septic myocardial damage, thus improving cardiomyocyte function (Liu et al., 2019a; An et al., 2016). For instance, Chen et al. observed significant alterations in the PI3K/AKT/mTOR signaling pathway while investigating the pathogenesis of SIMI (Chen et al., 2019). Additionally, Shang et al. demonstrated that resveratrol exerted protective effects on the myocardium of septic rats by activating the PI3K/AKT/mTOR signaling pathway, inhibiting the NF-κB signaling pathway, and suppressing inflammatory factors (Shang et al., 2019a). Similarly, in the lipopolysaccharide (LPS)-induced H9C2 cell model, rosmarinine ameliorated LPS-induced myocardial dysfunction by modulating the PI3K/AKT/mTOR pathway, thus exerting anti-apoptotic and anti-oxidative stress effects (Qi et al., 2021).

### 2.5 TLR/NF-κB signaling pathway

As mentioned earlier, NF-κB is composed of p65 and p50 subunits, which can form homo- or heterodimers. In the cytoplasm, NF-κB remains in an inactive state as it binds to the inhibitory protein IkB, forming a trimeric complex. Interestingly, when the upstream signaling factor TNF binds to cell surface receptors, it induces conformational changes in the receptor, leading to phosphorylation of IkB and subsequent dissociation of the trimeric complex. Subsequently, the NF-κB dimer exposes a nuclear localization sequence (NLS), allowing it to rapidly translocate from the cytoplasm into the nucleus. Once in the nucleus, the NF-κB dimer binds to specific sequences on nuclear DNA, thereby facilitating the transcription of relevant genes. Several studies have demonstrated that the NF-κB pathway is closely associated with the molecular mechanisms of SIMI-induced apoptosis. Recently, it has been discovered that the TLR-4/NF-κB-mediated signaling pathway is involved in sepsis-induced cardiomyocyte apoptosis (Li et al., 2015; Karra et al., 2015). During sepsis, LPS within the cell wall of Gram-negative bacteria can transduce extracellular signals by activating intrinsic immune recognition by TLR-4. This activation subsequently triggered a series of events, including the activation of IRAK1 and TRAF6. Upon activation, NF-κB quickly translocated from the cytoplasm to the nucleus, initiating the transcription of target genes and leading to the release of TNF-α and IL-6 (Zhou et al., 2016; Park et al., 2015). Previous studies revealed that TNF-α and IL-6 were involved in SIMI by inhibiting myocardial function through activation of intracellular signaling and apoptosis (Jiang et al., 2014; Hiram et al., 2014). Therefore, targeting the TLR-4/NF-κB signaling pathway may be a potential approach to counteract apoptosis and alleviate SIMI. Xie et al. conducted a study on a mouse model of LPS-induced septic cardiomyopathy and discovered that miR-146a played a negative feedback role in inhibiting the TLR-4/NF-κB signaling pathway. This, in turn, exerted antiapoptotic effects and ameliorated SIMI injury (Xie et al., 2019). Additionally, miR-146a was found to attenuate SIMI by inhibiting the NF-κB signaling pathway through targeted modulation of ErbB4 (An et al., 2018). It is worth noting that the TLR2/NF-κB-mediated signaling pathway may also be involved in SIMI. Inhibition of the TLR2/NF-κB signaling pathway has been shown to alleviate SIMI (Wang et al., 2019b).

### 2.6 Apoptosis-related proteins in SIMI

#### 2.6.1 Caspase family

The caspase family inhibits cell survival pathways and specifically activates related proapoptotic factors, which might play an indispensable role in cardiomyocyte apoptosis during sepsis. When sepsis triggers cardiomyocyte injury, on one hand, the exogenous apoptotic pathway is activated by the binding of ligands to death receptors, such as FAS. This binding leads to the recruitment, dimerization, and activation of caspase-8 with the assistance of bridging proteins like FAS-associated death domain (FADD) and TNF receptor type 1-associated death domain (TRADD). The activated caspase-8 then directly initiates apoptosis by cleaving and activating caspases (−3, −6, and −7), or it can induce the endogenous apoptotic pathway by cleaving BID. On the other hand, the endogenous or mitochondrial apoptotic pathway can be activated by various cytokines, resulting in the release of cytochrome C from mitochondria. This leads to the formation of apoptosome composed of APAF1, cytochrome C, ATP, and caspase-9, which can potentially activate caspase-9. The activated caspase-9 then initiates apoptosis through cleavage and activation of the effector caspase-3 and caspase-7.

In a mouse model of LPS-induced sepsis, Carlson et al. discovered that inhibiting caspase activity with the broad-spectrum caspase inhibitor Z-Val-Ala-Asp (OMe)-FMK significantly restored cardiomyocyte function. This study clearly demonstrated the involvement of TNF-α-dependent apoptotic cascade reactions in the development of LPS-induced cardiac dysfunction (Laster et al., 1988). In addition, in a Gα(q) transgenic mouse model, Hayakawa et al. observed improvements in cardiomyocyte function and a significant reduction in mortality after reducing caspase-3 activity using the multi-caspase inhibitor IDN-1965 (Hayakawa et al., 2003). The aforementioned findings suggested that increased activity of caspase-3, a critical protein in apoptosis, is implicated in SIMI. Furthermore, it has been observed that caspase activity related to apoptosis is elevated in SIMI (Nevière et al., 2001).

#### 2.6.2 Bcl-2 family

The Bcl-2 family is a pivotal protein family involved in the regulation of apoptosis. It comprises pro-apoptotic proteins such as BH3-Only and BH 1–3, as well as anti-apoptotic proteins like Bcl-2 and Bcl-w. These two classes of proteins cooperate during the process of cellular apoptosis, collectively influencing whether a cell undergoes programmed cell death through modulation of the signaling pathway within the mitochondria. In the classical animal model of SIMI, the miR-499-SOX6-PDCD4 signaling pathway played a role in regulating apoptosis by modulating the BCL-2 family. Specifically, miR-499 inhibited the expression of pro-apoptotic genes such as BAD, BAX, and BID by suppressing the activity of SOX6 and PDCD4. Additionally, it upregulated the expression of the anti-apoptotic gene BCL-XL (Jia et al., 2016). The current study indicated that the development of SIMI may be associated with the activation of Bax and the inhibition of Bcl-2, resulting in apoptosis in cardiomyocytes during sepsis (Rudiger and Singer, 2007). Li et al., 2022a discovered that miR-21 can promote apoptosis in SIMI by suppressing the expression of Bcl-2. On the other hand, IL-3 was found to inhibit apoptosis in SIMI by upregulating the expression of BCL-2 and downregulating the levels of BAX (Hu et al., 2020).

Based on the aforementioned studies, it can be postulated that inhibiting the expression of pro-apoptotic proteins and their associated pathways, as well as promoting the expression of anti-apoptotic proteins and pathways, are crucial therapeutic approaches for treating SIMI.

Previous studies have demonstrated that in SIMI mice, the apoptosis of cardiomyocytes can be inhibited by increasing the expression of Bcl-2 and reducing the expression of Bid, t-Bid, and caspase-9 (Xu et al., 2020a). Li et al., 2021a discovered that irisin can upregulate Bcl-2 levels, decrease the protein levels of Bax and its downstream effector caspase-3 in myocardial cells affected by sepsis, thereby inhibiting sepsis-induced apoptosis and treating SIMI. Shao et al. found that in SIMI mice, tensin reduced LPS-induced cardiomyocyte apoptosis, enhanced cell viability, downregulated the expression of caspase-1, IL-1β, and Bax, and upregulated the expression of Bcl-2 in cardiomyocytes (Shao et al., 2021). The aforementioned study not only confirmed that regulating key proteins involved in apoptosis could provide a new perspective for the treatment of septic cardiomyopathy but also suggested that one of the underlying mechanisms of SIMI may be the imbalance between the endogenous anti-apoptotic pathway mediated by Bcl-2 and the exogenous pro-apoptotic pathway involving caspase-8/Bid/t-Bid/caspase-9, as well as the imbalance between these two pathways. This provides further insights into the pathogenesis of SIMI. Note, caspase-8 not only played a crucial role in apoptosis but also contributed to RIPK1/RIPK2/MLKL-mediated necroptosis in the molecular mechanism of septic cardiomyopathy (Feoktistova et al., 2011). Therefore, we believed that cardiomyocyte apoptosis, necroptosis, and other forms of programmed cell death are not independent but rather interconnected interact with each other, collectively resulting in SIMI.

Currently, research on apoptosis-related proteins in SIMI primarily revolves around the caspase family and the Bcl-2 family. However, the precise roles and mechanisms of other apoptosis-related proteins in SIMI remain unclear. Therefore, further investigation is necessary to deepen our understanding of the apoptotic mechanisms involved in SIMI.

To summarize, apoptosis plays a crucial role in the mechanism of SIMI. Blocking the apoptosis signaling pathway and inhibiting the expression and activity of apoptosis-related proteins may potentially alleviate SIMI. Whereas, further exploration of the apoptosis mechanism in SIMI is necessary to provide a valuable theoretical basis for targeted therapy in the future.

## 3 Necroptosis in SIMI

Necroptosis is a novel mechanism of PCD that can display both necrotic and apoptotic characteristics, as evidenced by cellular swelling, membrane rupture, chromosome condensation, as well as the release of damage-associated molecular patterns (DAMPs), inflammatory cytokines, and chemokines that contribute to intense inflammatory responses. Multiple studies have indicated necroptosis might be indispensable in intrinsic mechanisms underlying the release of inflammatory factors and the development of infectious diseases (Han et al., 2011; Moreno-Gonzalez et al., 2016). Additionally, the pathogenesis of sepsis may involve a profound and persistent inflammatory response resulting from the systemic activation of the immune system by invading microorganisms (Delano and Ward, 2016; Ehrman et al., 2018). Therefore, we can regard necroptosis as an initial “storm of cell death” that can result in acute or persistent inflammatory responses. Targeting the molecular mediators of necroptosis could represent a novel and effective strategy for treating sepsis and the associated organ damage. Therefore, it is crucial to elucidate the pathways, key proteins, and classical signaling pathways involved in necroptosis in SIMI to develop effective clinical therapeutic strategies for SIMI and enhance cardiomyocyte function. In the following sections, we will discuss necroptosis in SIMI from the aforementioned three aspects.

### 3.1 Pathways of necroptosis in SIMI

**FIGURE 4:**
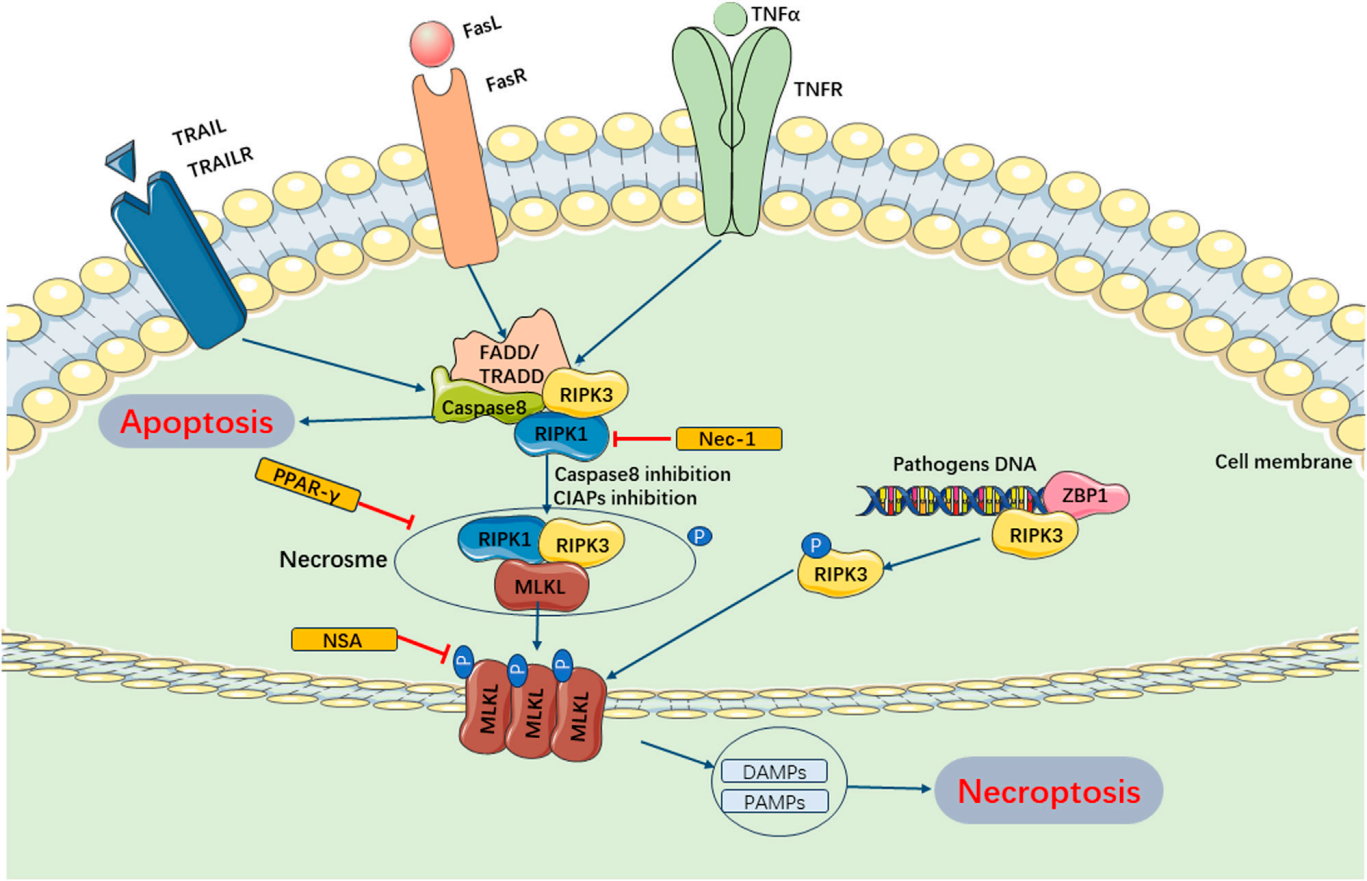
Main Pathways of Necroptosis. Necroptosis can be mainly regulated by RIPK1 and RIPK3, which can form necrosome and inhibition of caspase-8 and cIAPs. The necrosome trigger phosphorylation of MLKL and enables it to form pores in the membrane. These MLKL pores allows for ion efflux, cellular swelling, membrane rupture, and subsequent uncontrolled release of intracellular contents, ultimately resulting in necroptosis. Furthermore, pathogenic microorganisms can release cytoplasmic DNA and combine ZBPI, recruiting RIPK3, activating MLKL and forming necrotic complex, which contributes to necroptosis. cIAPs, cellular inhibitor of apoptosis proteins; TRAIL, TNF-related apoptosis-inducing ligand; TRAILR, receptor of TNF-related apoptosis-inducing ligand; ZBPI, Z-DNA binding protein 1; DAMPs, damage-associated molecular patterns; PAMPs, pathogen-associated molecular patterns.

Current studies have revealed that necroptosis is primarily activated downstream of death domain receptors (e.g., TNFR and Fas) and Toll-like receptor (TLR)-4 or TLR3 during the progression of SIMI (Laster et al., 1988; Holler et al., 2000; He et al., 2011). Upon ligand binding, these receptors recruited adaptor proteins such as FADD, TRADD, and TRIF, leading to a cascade of changes upon interaction with receptor interacting protein-1 (RIPK1) and caspase −8 or −10 ^63^- (Tenev et al., 2011). Under normal circumstances, RIPK1 is ubiquitinated by IAPs to maintain its non-functional state. Upon detection of a death signal, RIPK1 underwent deubiquitinating by CYLD, leading to the recruitment of RIPK3 and formation of the RIPK1/RIPK3 complex (Martin and Ranieri, 2011a; Li et al., 2012). The RIPK1/RIPK3 complex recruited and phosphorylated mixed lineage kinase domain-like (MLKL) (Zhao et al., 2012). Subsequently, MLKL underwent oligomerization to form necroptotic vesicles, which then generate larger channels called MLKL pores on the plasma membrane. These MLKL pores allowed for ion efflux, cellular swelling, membrane rupture, and subsequent uncontrolled release of intracellular contents, ultimately resulting in necroptosis (Murphy et al., 2013). Additionally, it has been discovered that if pathogenic microorganisms can release cytoplasmic DNA, the DNA-dependent activator of IFN regulatory factor (DAI) also recruits RIPK3, bypassing RIPK1 activation and leading to the activation of MLKL and formation of the necrotic complex, which contributes to necroptosis (Upton et al., 2012; Maelfait et al., 2017). Current evidences have suggested that necroptosis plays a role in the pathogenesis of SIMI. Zhang et al. (2020) demonstrated that rosiglitazone improved myocardial function in rats with septic cardiomyopathy by weakening TNFR-triggered necroptosis (Figure 4).

### 3.2 Classical signaling pathways of necroptosis in SIMI

In SIMI, the RIPK1/RIPK3/MLKL signaling pathway is considered as the canonical pathway involved in necroptosis, particularly in TNFα-mediated necroptosis. The previous text has already introduced the three essential proteins involved in this signaling pathway. Upon activation of TNFα, RIPK1 undergoes phosphorylation and recruits RIPK3 to form a complex, subsequently recruiting MLKL to assemble necrosomes, which ultimately triggers necroptosis.

Studies have suggested a significant increase in the intracellular expression of MLKL and RIPK3 in patients with SIMI compared to healthy controls (Mallarpu et al., 2021). Nevertheless, it is not appropriate to generalize that necroptosis has universally negative effects on all organisms. For instance, necroptosis also played a crucial role in eliminating excessive activated lymphocytes in sepsis, which was critical for maintaining lymphocyte homeostasis (Bland et al., 1976). Additionally, eukaryotic cells may undergo necroptosis as a defense mechanism following bacterial and viral infections to inhibit pathogen replication and indirectly protect vital organs in sepsis (Kitur et al., 2016).

### 3.3 Necroptosis-related proteins in SIMI

In SIMI, the key proteins involved in necroptosis are RIPK1, RIPK3, and MLKL. Both RIPK1 and RIPK3 are serine/threonine kinases that play critical roles in mediating necroptosis. Clinical studies have revealed that RIPK3 levels in patients with severe sepsis and septic shock are significantly elevated compared to the sepsis group at all time points. These elevated levels were positively correlated with Sequential Organ Failure Assessment (SOFA) scores and procalcitonin (PCT) levels (Wang et al., 2017). Recent clinical trials have further demonstrated that necroptosis can serve as a predictor of mortality in sepsis patients, and RIPK3 levels can be utilized as a marker for assessing necroptosis (Mallarpu et al., 2021). Based on clinical trials, molecular mechanism studies have revealed that RIPK3, a marker of necroptosis, is positively associated with mortality and organ dysfunction in sepsis (Schenck et al., 2019). Furthermore, in various injury models, Necrostatin-1 (Nec-1) has been shown to inhibit necroptosis by blocking the activity of RIPK1 kinase, suggesting its potential therapeutic role in disease management (Rosenbaum et al., 2010; Kaczmarek et al., 2013). In the cecal ligation and puncture (CLP) sepsis model, Peng et al., 2017 demonstrated that activation of PPAR-γ reduced the expression of RIPK1, RIPK3, and its downstream effector MLKL, thereby inhibiting necroptosis and improving myocardial function in sepsis. MLKL, as a downstream effector of the RIPK1/RIPK3/MLKL signaling pathway, can be considered as a direct executor of necroptosis. Once activated, MLKL translocated to the cell membrane, leading to its disruption and release of danger signals (Tang et al., 2019a). In the LPS-induced sepsis model, inhibition of MLKL activity with necrosulfonamide (NSA) markedly attenuated necroptosis (Rathkey et al., 2018).

In summary, the role of necroptosis in sepsis and SIMI remains inconsistent, and the underlying mechanism has not been fully elucidated. Further investigation is warranted to explore this topic in future studies.

## 4 Pyroptosis in SIMI

Pyroptosis, also known as caspase-1-dependent cell death, is a PCD process that occurs when there is a disruption in intracellular or extracellular homeostasis. It is closely associated with innate immunity. Pyroptosis is primarily mediated by the formation of plasma membrane pores by members of the gasdermin (GSMD) protein family, leading to the release of various inflammatory factors such as IL-1 and IL-18 ^86^. This process is characterized by the formation of pores on the cell membrane, cellular swelling and rupture, accompanied by the release of a significant number of inflammatory factors and cellular contents (Cookson and Brennan, 2001; Galluzzi et al., 2018). Pyroptosis is closely associated with the massive release of inflammatory factors, which can trigger sepsis and SIMI. Therefore, pyroptosis is believed to be relevant to the development of sepsis and septic organ damage, making it a significant research focus in the field. Kalbitz et al., 2016 observed a significant increase in levels of NLRP3 and IL-1β in left ventricular cardiomyocytes of mice with sepsis induced by cecal ligation and puncture (CLP). When the NLRP3 gene was knocked down in mice, they exhibited lower levels of cardiovascular injury and plasma IL-1β and IL-6 compared to wild-type mice. These findings suggested the involvement of NLRP3 in SIMI. Similarly, other studies have demonstrated improved survival rates and cardiac function in septic mice following NLRP3 gene knockout (Busch et al., 2021). This review aims to provide molecular evidence for pyroptosis as a therapeutic target for SIMI by summarizing the role of pyroptosis in SIMI from three aspects.

### 4.1 Pathways of pyroptosis in SIMI

**FIGURE 5:**
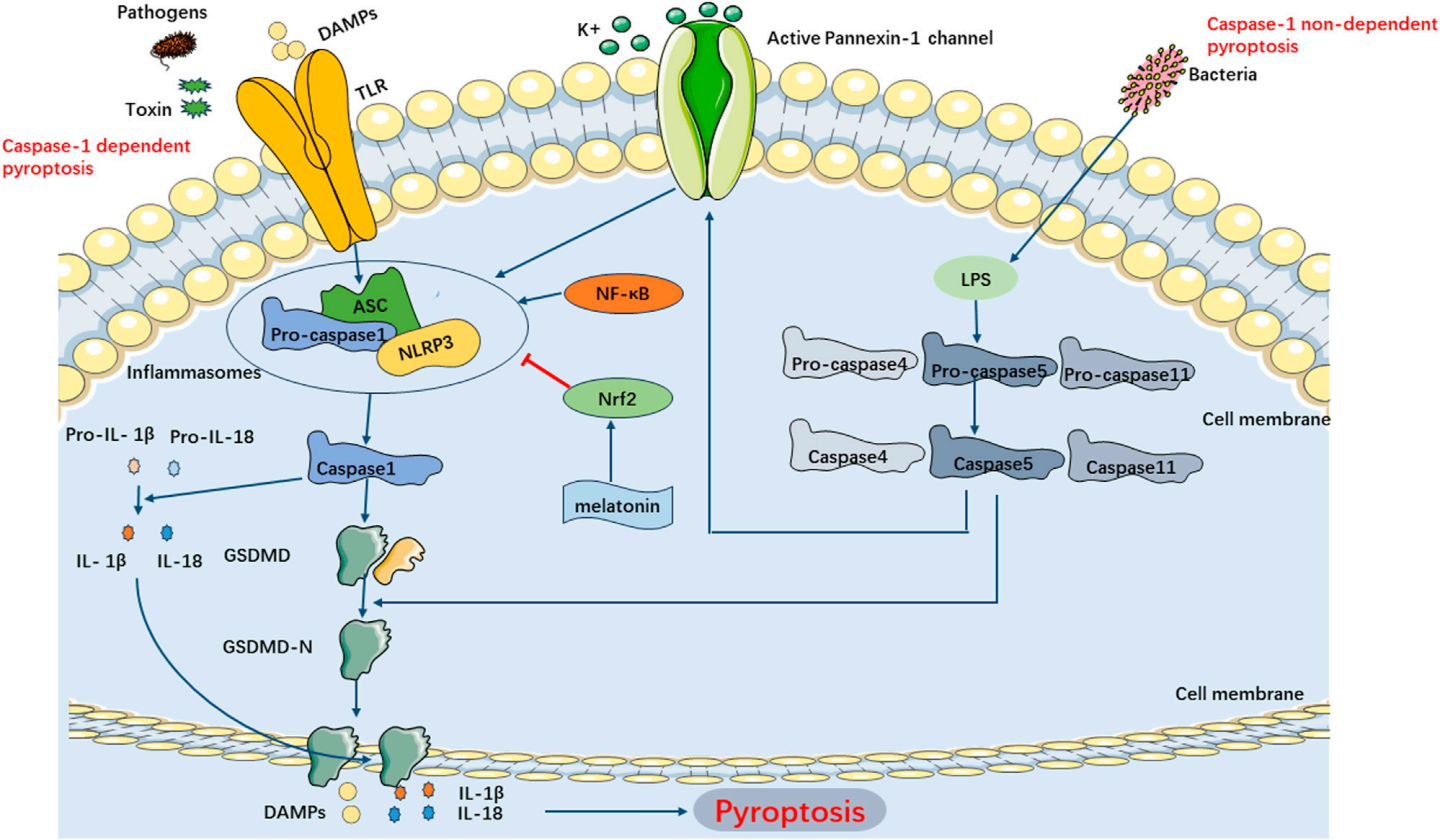
Main Pathways of Pyroptosis. Pyroptosis can be categorized into two types depending on whether they are caspase1 dependent or not. In caspase-1 dependent pyroptosis, the process is initiated by the assembly of inflammasomes. In caspase-1 non-dependent pyroptosis can be triggered by the interaction between caspase4, caspase5, or caspase11 (depending on the species) and LPS.

The classical pyroptosis pathway in SIMI can be categorized into two types depending on whether they are caspase-1 dependent or not. The caspase-1-dependent pyroptosis pathway is as follows: Upon invasion of various pathogens into cells, inflammatory vesicles such as NLRP3, NLRC4, AIM2, Pyrin, etc., recognize the signals and become activated, subsequently activating caspase-1 through the binding of adaptor protein ASC to Pro-Caspase-1. On one hand, activated caspase-1 cleaves GSDMD, exposing the N-terminus of GSDMD, which binds to phospholipoproteins on the cell membrane, resulting in the formation of pores. These pores lead to the release of cellular contents, triggering the onset of pyroptosis. On the other hand, activated caspase-1 also cleaves and activates the precursors of IL-1β and IL-18. The activated forms of IL-1β and IL-18 are then released extracellularly, thereby amplifying the inflammatory response. The caspase-1 non-dependent pyroptosis pathway in SIMI is initiated as follows: Upon stimulation with LPS, caspase-4, caspase-5, and caspase-11 directly bind and activate with LPS. This activation leads to the cleavage of GSDMD proteins, exposing their N-termini, thereby initiating the pyroptotic process. Additionally, the activated caspase-4/5/11 phosphorylates the Pannexin-1 channel and releases K+ extracellularly, which activates NLRP3 inflammasomes, leading to caspase-1 activation and further activation of the caspase-1-dependent pyroptosis pathway (Liu et al., 2016) (Figure 5).

### 4.2 Classical signaling pathways of pyroptosis in SIMI

The ER/SIRT1/NLRP3/GSDMD signaling pathway is a classical signaling pathway associated with pyroptosis in the pathogenesis of SIMI (Huang et al., 2022). It is currently considered the most comprehensive pathway discovered to date. Inhibiting the aforementioned signaling pathway to impede pyroptosis and improve cardiomyocyte function holds promise as a potential therapeutic target for SIMI. Transcription factors have been discovered to participate in the regulation of pyroptosis in sepsis by modulating this pathway. NF-κB, for instance, may play a crucial role in the initial activation of the NLRP3 inflammasome by upregulating the transcriptional expression of NLRP3 and pro-IL-1β, thereby ensuring the activation and assembly of the NLRP3 inflammasome (Wen et al., 2022). Furthermore, p65, a member of the transcription factor family, can directly bind to the NLRP3 promoter, suggesting its direct involvement in regulating NLRP3 expression in LPS-induced brain microvascular endothelial cells (BMECs) (Chen et al., 2020a). Nuclear factor erythroid 2-related factor (Nrf2) is a transcription factor that regulates the cellular response to oxidative stress and the expression of various cytoprotective genes, thereby maintaining redox homeostasis. Studies have shown that downregulation of Nrf2 facilitates pyroptosis in sepsis (Pu et al., 2017; Li et al., 2022b). In addition, Rahim et al. demonstrated that melatonin ameliorated sepsis-induced myocardial injury by activating Nrf2-related pathways and inhibiting the formation of NLRP3 inflammasomes (Rahim et al., 2021). Furthermore, Nrf2 can upregulate the expression of C1q/tumor necrosis factor-related protein 1 (CTRP1) by binding to its promoter, thereby inhibiting pyroptosis and alleviating myocardial injury in sepsis (Teng et al., 2022). These findings suggest that Nrf2 can protect cardiomyocytes from sepsis-induced injury by suppressing pyroptosis.

### 4.3 Pyroptosis-related proteins in SIMI

The hallmark events of pyroptosis include the activation of NLRP3 inflammatory vesicles, formation of GSDMD pores, and secretion of pro-inflammatory cytokines. As the first identified executor of pyroptosis, GSDMD acts downstream of caspases in response to cellular inflammatory regulation, triggering pyroptosis (Vande Walle and Lamkanfi, 2016). In a septic mouse model, levels of GSDMD-nt, cleaved caspase-1, and pro-inflammatory cytokines were significantly elevated (Fu et al., 2019). The NLRP3 inflammasome plays a crucial role in the mechanism of pyroptosis in sepsis. Previous clinical studies have reported elevated levels of NLRP3, GSDMD, IL-1β, and IL-18 in patients with sepsis (Dolinay et al., 2012; Homsy et al., 2019; Huang et al., 2022). Building upon previous research on the cardioprotective effects of rhodopsin, it has been suggested that the activation of NLRP3 inflammasomes triggers pyroptosis in septic cardiomyocytes. This indirect regulation of pyroptosis by the NLRP3 inflammasome occurs through the caspase-1, ROS, and NF-κB signaling pathways (Zhaolin et al., 2019). Furthermore, rhodopsin has been shown to decline the expression of NLRP3 and GSDMD in myocardial tissues, suppressing the activation of the NLRP3 inflammasome and ultimately reducing septic myocardial injury in an LPS-induced sepsis model (Dai et al., 2021).

In conclusion, pyroptosis is a critical factor in the mechanism of SIMI. Considering pyroptosis as a potential therapeutic target for the treatment of SIMI is feasible in the future.

## 5 Ferroptosis in SIMI

Ferroptosis is a novel form of PCD that is characterized by its iron and reactive oxygen species (ROS) dependency. The imbalance between ROS and antioxidants can trigger oxidative stress and activate various pro-inflammatory factors, including NF-κB and hypoxia-inducible factor-1 (Yu et al., 2021). In addition to being essential for ferroptosis, ROS is also necessary for the activation of NLRP3. Previous studies have demonstrated the critical role of Nrf2 in both pyroptosis and ferroptosis (Pu et al., 2017; Li et al., 2022b; Wang et al., 2022a), suggesting a potential connection between these two processes (Arioz et al., 2019). The essence of ferroptosis is the depletion of glutathione. Specifically, the activity of glutathione peroxidase 4 (GPX4) is diminished, leading to the inability of lipid peroxides to be metabolized through the GPX4-catalyzed glutathione reductase reaction. Consequently, Fe2+ oxidizes lipids, resulting in the production of reactive oxygen species and ultimately contributing to ferroptosis. In short, the initiation and progression of ferroptosis are influenced by various metabolic processes, including iron homeostasis, amino acid metabolism, and lipid peroxidation. Furthermore, other molecules like coenzyme Q can also participate in regulating the sensitivity of ferroptosis (Qu et al., 2021). To date, the most prominent feature of ferroptosis is the generation of lipid peroxides, which subsequently leads to cell membrane damage (Tang et al., 2019a). Ferroptosis may participate in the pathophysiological processes of various organs, including the nervous, urinary, hepatic, and cardiovascular systems (Fang et al., 2019; Gao et al., 2015; Xie et al., 2016). Recent studies have confirmed the involvement of ferroptosis in the onset and progression of sepsis (Zhu et al., 2019). Morphological characteristics of ferroptosis may include mitochondrial shrinkage, decreased mitochondrial ridges, increased cell density, outer membrane rupture, along with an unaltered nucleus (Jiang et al., 2021; Beatty et al., 2021). Due to the high mitochondrial content in myocardial tissue, previous studies have primarily investigated ferroptosis in septic myocardial injury, compared to other organs such as the renal and nervous systems (Friedmann Angeli et al., 2014; Gao and Chang, 2014). Wang et al., 2020b demonstrated that dexmedetomidine may exert a protective effect in septic myocardial injury by inhibiting ferroptosis through α2-AR activation. This finding suggests that ferroptosis might play a critical role in the mechanism of septic myocardial injury. Similar to other forms of PCD mentioned above, this section will discuss the ferroptosis of SIMI from the following three aspects.

### 5.1 Pathways of ferroptosis in SIMI

**FIGURE 6:**
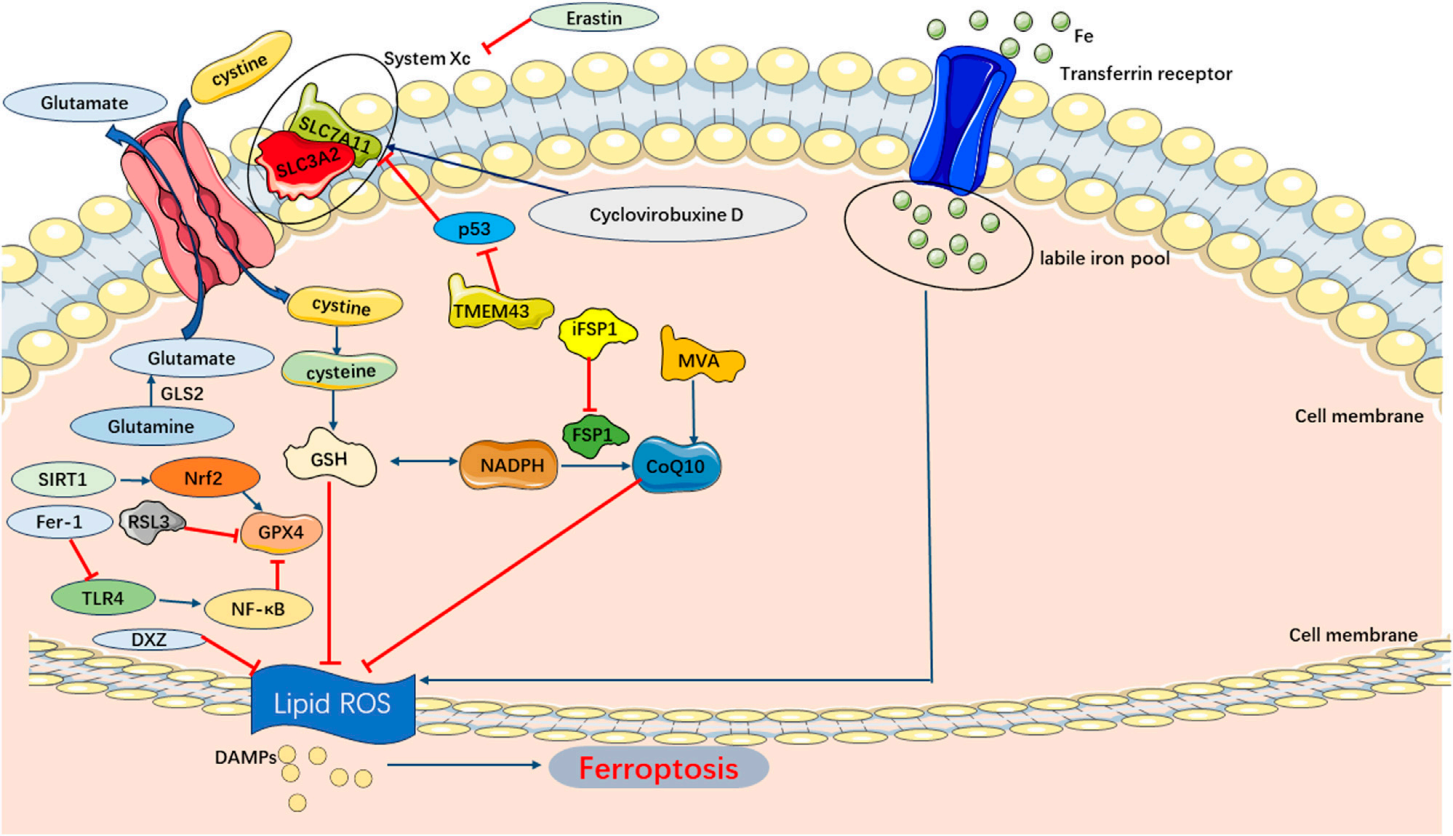
Main Pathways of Ferroptosis. Cystine is transported into the cell by System Xc-for synthesis of GSH, which can be used by GPX4 as a substrate to prevent lipid ROS accumulation. And lipid peroxidation also could be inhibited by CoQ10 generated from MVA. Consequently, the accumulation of lipid ROS triggers ferroptosis.

Ferroptosis pathways in SIMI predominantly involve the exogenous transporter-dependent and endogenous/enzyme-regulated pathways. In the exogenous/transporter-dependent pathway, cellular responses to infection, stress, and inflammation lead to the inhibition of cell membrane transporters such as the cystine/glutamate reverse transporter (System xc-). The physiological function of System xc-is to facilitate the inward transport of extracellular cystine and participate in glutathione synthesis. Glutathione serves as a reductive cofactor for glutathione peroxidase 4 (GPX4). When System xc-is inhibited, the synthesis of glutathione is impaired, thereby reducing the activity of the membrane lipid repair enzyme GPX4. Consequently, cellular antioxidant capacity is diminished, ultimately triggering ferroptosis. Furthermore, activation of iron transporters, specifically serum transferrin and lactotransferrin, can also induce ferroptosis (Bergmann et al., 2009; Dixon et al., 2012). In the endogenous/enzyme-regulated pathways, ferroptosis can occur by suppressing the activity of intracellular antioxidant enzymes, primarily including glutathione peroxidase 4 (GPX4), ferroptosis suppressor protein 1 (FSP1), and coenzyme Q10 (FSP1/CoQ/NADPH) (Bergmann et al., 2009) (Figure 6).

### 5.2 Classical signaling pathways of ferroptosis in SIMI

Current studies on the mechanism of ferroptosis in SIMI primarily focus on the Sirt1/Nrf2/GPX4 signaling pathway. Previous studies suggested that the activation of this signaling pathway can inhibit ferroptosis and attenuate cardiac dysfunction (Dodson et al., 2019). Sirtuin 1 (SIRT1), as a member of the sirtuin family, is an NAD + -dependent histone deacetylase involved in various biological processes, including cellular metabolism, aging, inflammation, and oxidative stress (Hwang et al., 2013; Singh and Ubaid, 2020a). SIRT1 can activate the transcription factor Nrf2, and activated Nrf2 exerts anti-inflammatory, antioxidant, and cytoprotective effects (Arioz et al., 2019; Zhang et al., 2018; Singh and Ubaid, 2020b). To date, numerous studies have demonstrated that Nrf2 is involved in maintaining the stability of iron metabolism in the body. For instance, Nrf2 can be involved in the storage and transportation of iron ions by regulating the light chain of ferritin (FTL/FTH1) and the heavy chain of iron transport protein (SLC40A1), respectively (Sun et al., 2022; Harada et al., 2011). In a septic cardiomyopathy model, the inactivation of Sirt1 and Nrf2, along with decreased GPX4 expression, can promote ferroptosis and worsen cardiomyocyte injury (Wang et al., 2022a). However, further treatment with RSV in the septic cardiomyopathy model can positively regulate the molecular mechanism of Sirt1/Nrf2/GPX4 signal transduction. Nrf2 can induce the expression of SLC7A11 and GPX4 in the GSH antioxidant system, enhancing the antioxidant capacity of cardiomyocytes, suppressing ferroptosis, and ultimately alleviating cardiac depression (Wang et al., 2022a). Similarly, NaHS has been found to alleviate septic myocardial injury and inhibit ferroptosis by reducing BECN1 phosphorylation and subsequently upregulating the expression of GPX4 and SLC7A11 (Cao et al., 2022).

Notably, the Sirt1/Nrf2/GPX4 pathway was also involved in mechanisms of septic target organ damage other than the heart. For instance, irisin may attenuate the inflammatory microenvironment in septic encephalopathy by restraining hippocampal ferroptosis through the Nrf2/GPX4 signaling pathway (Wang et al., 2022b). In addition to the Sirt1/Nrf2/GPX4 pathway, there are other regulatory signaling pathways that can influence ferroptosis in SIMI. For example, solute carrier family 7 member 11 (SLC7A11) underwent N6-methyladenosine (m6A) methylation, which can be recognized and degraded by YTHDF2, thereby facilitating ferroptosis in SIMI (Shen et al., 2023). Furthermore, the TLR4/NF-κB signaling pathway is considered to regulate ferroptosis. Xiao et al. discovered that ferrostatin-1 (Fer-1) can downregulate the levels of TLR4 and phosphorylated NF-κB, suppressing TLR4/NF-κB signaling pathway, ultimately inhibiting ferroptosis and alleviating cardiomyocytes (Xiao et al., 2021).

### 5.3 Ferroptosis-related proteins in SIMI

GPX4 is a member of the selenium-containing GPX family and demonstrates the ability to scavenge membrane lipid hydroperoxide products. Physiologically, GPX4 utilizes its catalytic activity to mitigate the toxicity of lipid peroxides and maintain homeostasis in the membrane lipid bilayer. By inhibiting the production of ROS, GPX4 can effectively prevent the occurrence of ferroptosis. When GPX4 is deficient in synthesis or its activity is inhibited, accumulation of intracellular peroxides occurs, leading to ferroptosis (Stockwell et al., 2017). Studies have further demonstrated that RSL3 inhibits the activity of GPX4, resulting in a decrease in cellular antioxidant capacity and an increase in levels of reactive oxygen species derived from lipid metabolism, ultimately leading to ferroptosis (Lv et al., 2023). Additionally, selenocysteine, an amino acid present in the active site of GPX4, is influenced by the mevalonate pathway (MVA pathway). This pathway can downregulate the production of isopentenyl pyrophosphate (IPP), which subsequently negatively regulates the maturation of selenocysteine transfer RNA, ultimately resulting in the inhibition of GPX4 activity and the induction of ferroptosis (Yu et al., 2017). Recent studies have provided evidence for the role of ROS in sepsis development, leading to the induction of ferroptosis (Bogdan et al., 2016). In a mouse model of sepsis induced by LPS, LPS administration upregulated the expression of markers associated with iron-triggered damage, such as prostaglandin endoperoxide synthase 2 (PTGS2), malondialdehyde (MDA), and lipid ROS. This induction of ferroptosis resulted in mitochondrial damage, which was attenuated by the administration of fe-1and DXZ (Li et al., 2020). Furthermore, ferroptosis, involving the participation of GPX4, plays a crucial role in the pathophysiological processes of cardiomyocytes. Overexpression of GPX4 delayed palmitate-induced ferroptosis in cardiomyocytes, whereas the protective effect against ferroptosis was significantly weakened when GPX4 expression was low (Wang et al., 2021a). Another key protein in ferroptosis is the cystine/glutamate antiporter system Xc (also known as xCT), which consists of the catalytic subunit solute carrier family 7 member 11 (SLC7A11) and the chaperone subunit solute carrier family 3 member 2 (SLC3A2). Its primary function is to transport extracellular cystine into the cell, participating in glutathione formation and antioxidant defense (Koppula et al., 2021). p53 has an impact on ferroptosis through its regulation of SLC7A11. Jiang et al. have indicated that p53 can downregulate the expression of SLC7A11, leading to the inhibition of cystine uptake by SLC7A11. This inhibition results in a decrease in cystine-dependent glutathione peroxidase activity and cellular antioxidant capacity, ultimately triggering an elevation in lipid reactive oxygen species (LROS) and the occurrence of ferroptosis (Jiang et al., 2021). In addition, Erastin, which is a specific inducer of ferroptosis, reduces intracellular glutathione (GSH) levels and triggers ferroptosis by inhibiting the activity of SLC7A11^86^. In an LPS-induced SIMI mouse model, TMEM43 has been found to protect against SIMI by inhibiting p53 expression, increasing the levels of GPX4 and SLC7A11, and suppressing ferroptosis (Chen et al., 2022a). Furthermore, SLC7A11, as a major intracellular antioxidant glutathione (GSH) provider, can suppress lipid peroxidation and exert a protective role in both LPS-induced and non-LPS-induced cardiac injury through the mechanisms of ferroptosis. Zhang et al. discovered that SLC7A11 could prevent cardiac hypertrophy by inhibiting ferroptosis (Zhang et al., 2018). In an LPS-induced sepsis mouse model, Cyclovirobuxine D upregulated the expression of SLC7A11, attenuated ferroptosis, and ameliorated septic cardiomyocyte injury (Wang et al., 2023a). Apart from GPX4 and SLC7A11, there are other molecules that may contribute to ferroptosis, such as Heme oxygenase-1 (HO-1), which is involved in intracellular iron metabolism. Previous study demonstrated that HO-1 can induce lipid peroxidation and thereby promote the occurrence of ferroptosis (Han et al., 2022).

Currently, multiple studies suggested that inhibiting ferroptosis can alleviate SIMI. Nevertheless, some studies have also pointed out that ferroptosis can protect cells from inflammatory injury in certain situations. Zhou et al. discovered that Puerarin can alleviate septic myocardial injury through AMPK-mediated ferroptosis (Zhou et al., 2022). Therefore, it is notable that ferroptosis plays a dual role in cellular pathophysiological processes (Arbiser et al., 2018).

In summary, the specific mechanism of ferroptosis in SIMI has not yet been clarified. There is still ample room for further exploration in the treatment of SIMI, particularly regarding the regulation of ferroptosis and its dual role.

## 6 Autophagy in SIMI

**FIGURE 7:**
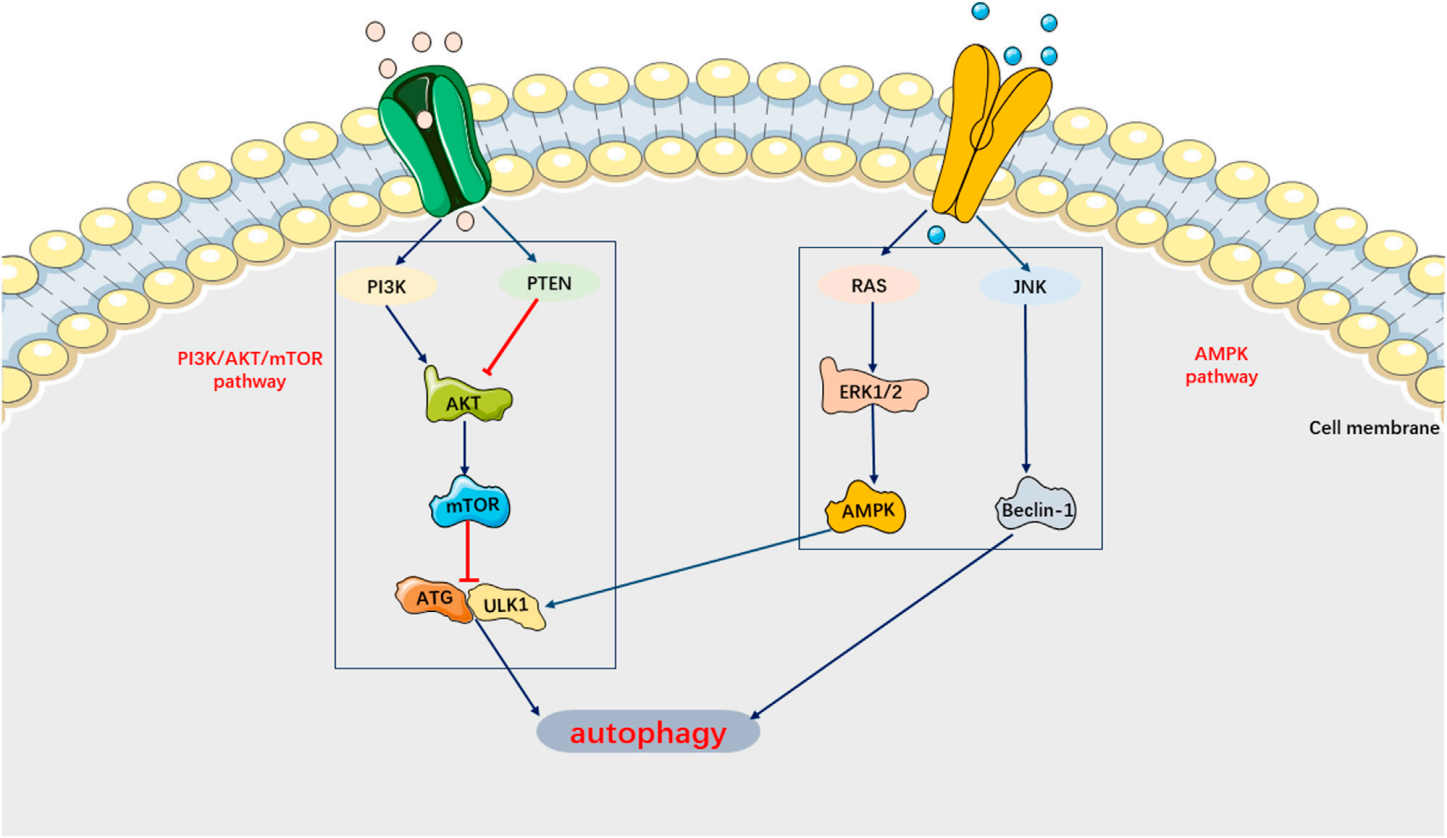
Main Signaling pathways of Autophagy. AMPK pathway can activate autophagy. PI3K/AKT/mTOR pathway can inhibit autophagy.

Autophagy can be activated to degrade cellular components, proteins, and damaged organelles in a lysosome-dependent manner, thereby preventing the spread of biomolecules and damaged organelles (Anand et al., 2020). Recent studies have indicated that autophagy protects against sepsis-induced damage to multiple target organs by activating macrophages and suppressing inflammatory factors (Jiang et al., 2015; Chung et al., 2017; Jia et al., 2019; Cui et al., 2019; Lu et al., 2019; Oami et al., 2017). However, in certain circumstances, autophagy can induce cell death (Denton and Kumar, 2019). For example, activation of autophagy has been found to promote the aggregation of granulocytes and other inflammatory cells, impair the phagocytic ability of macrophages and granulocytes against pathogenic bacteria, and exacerbate septic lung injury (Patoli et al., 2020). Therefore, autophagy may have a dual role in the pathophysiological processes of the organism. Previous studies have demonstrated that autophagy also plays a role in SIMI (Marek-Iannucci et al., 2021). Autophagy activation was found to mitigate cardiomyocyte damage induced by sepsis and contribute to the restoration of cardiac physiological function (Jia et al., 2019; Zhao et al., 2017). In other words, autophagy inhibition plays a crucial role in the mechanism of SIMI. However, recent studies have shown that inhibiting autophagy can still improve SIMI (Luo et al., 2020). For instance, estrogen and ulinastatin have been found to alleviate LPS-induced cardiac dysfunction by inhibiting autophagy (Wang et al., 2014; Zhao et al., 2020). Current basic research tends to support the notion that cells undergo dysregulated autophagy, leading to pathological injury (Klionsky et al., 2021). In SIMI, autophagy in cardiomyocytes is inhibited. Recent studies on autophagy in SIMI have primarily focused on the signaling pathways, specifically the AMPK pathway, which promotes autophagy, and the PI3K/AKT pathway and mTOR pathway, which inhibit autophagy (Figure 7).

On the one hand, AMPK, as an autophagy-initiating kinase, can activate ULK1 by phosphorylating Ser 317 and Ser 777, thereby initiating autophagy and inhibiting SIMI (Wu et al., 2020; Di et al., 2020). Studies have shown that impaired AMPK phosphorylation can lead to inhibited autophagy and worsened septic myocardial injury (Mao et al., 2021). On the other hand, negative stimuli such as cell injury can activate the PI3K/AKT/Bcl-2 pathway to suppress autophagy and exacerbate sepsis (Yuan and Wang, 2020). Similarly, inhibition of the mTOR signaling pathway facilitates autophagy, ultimately alleviating cardiomyocyte dysfunction in sepsis (Patoli et al., 2020; Sang et al., 2020). Indeed, previous studies have demonstrated that platelet-derived exosomes can enhance the production of neutrophil extracellular traps (NETs) in sepsis by activating the autophagy-associated AKT/mTOR signaling pathway. This process may contribute to the development of SIMI (Kaplan and Radic, 2012). Furthermore, the number of NETs has been found to be positively correlated with the severity of SIMI. Currently, it is believed that the mechanism underlying the autophagic response in SIMI may involve ULK1 phosphorylation, which is regulated by AMPK activation and mTOR inhibition.

The current studies have shown that activation of cardiomyocyte autophagy can attenuate cardiac dysfunction triggered by SIMI (Yuan et al., 2020; Zhang et al., 2019). Therefore, targeting autophagy may be a potential direction for SIMI treatment. Hsieh et al., 2011 demonstrated that inducing autophagy with rapamycin in a SIMI mouse model could mitigate cardiac dysfunction. Previous studies have also indicated that Sirtuin6 (SIRT6) can activate autophagy and alleviate myocardial depression in sepsis (Tasselli et al., 2017). Additionally, it has been observed that modulation of JNK signaling pathway-dependent autophagy can alleviate sepsis-induced cardiac systolic dysfunction (Wang et al., 2019a). As previously mentioned, the JNK signaling pathway also plays a role in apoptosis associated with SIMI, suggesting a potential connection between apoptosis and autophagy. However, the current understanding of the underlying mechanism of autophagy in SIMI is still lacking. Further comprehensive research is warranted to explore additional evidence for the clinical therapeutic strategies targeting SIMI.

## 7 Interaction of PCD pathways in SIMI

**FIGURE 8:**
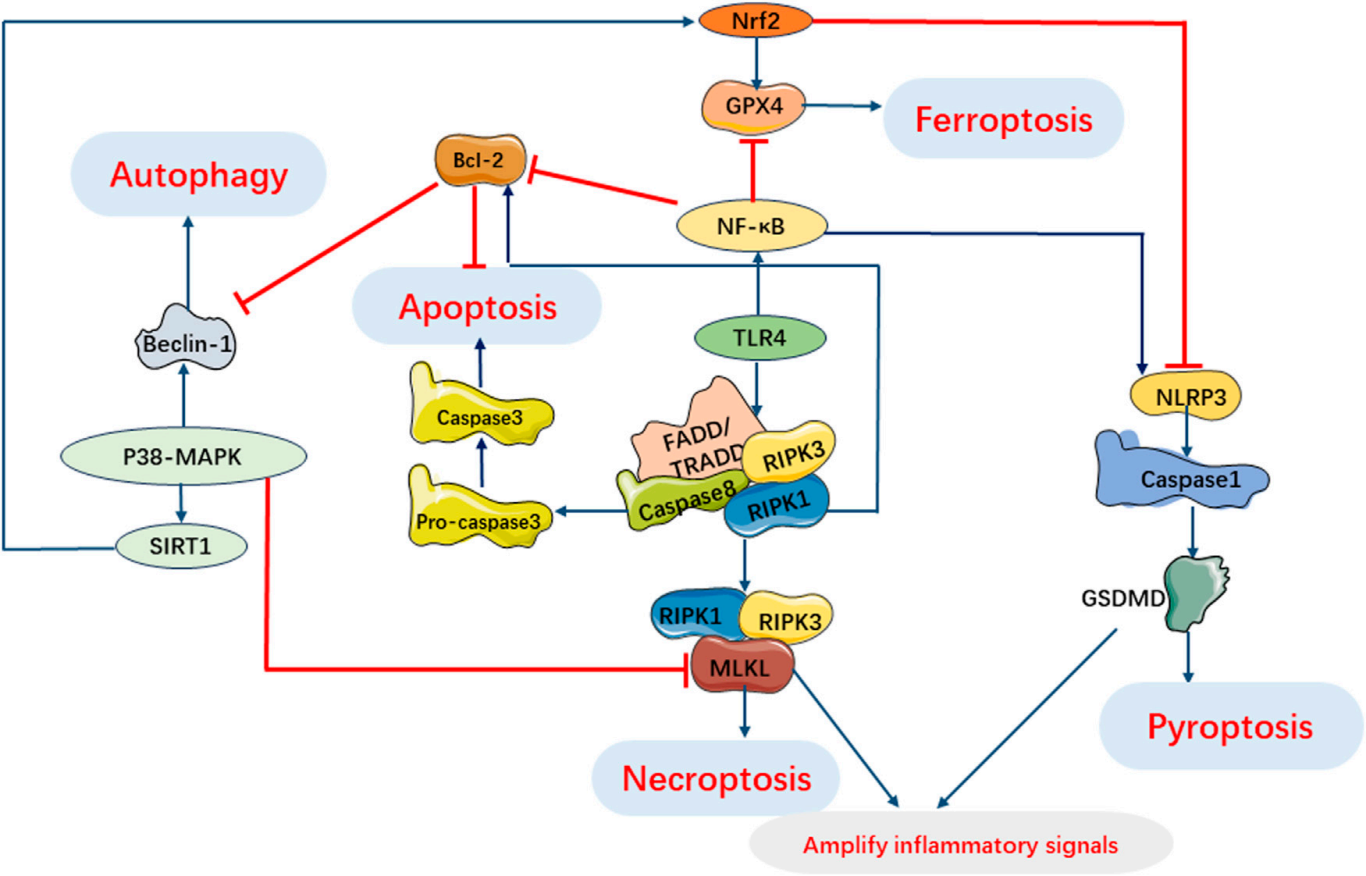
Interaction of Programmed Cell Death Pathways in SIMI. TLR4 involves pyroptosis, apoptosis and necroptosis. Activation of RIPK1 regulates necroptosis and induces apoptosis under oxidative stress and inflammatory processes. Beclin-1 regulates autophagy and apoptosis during infection. P38 MAPK pathway can inhibit necroptosis by inhibiting MLKL activation and regulates autophagy, ferroptosis and pyroptosis by regulating expression of SIRT1. NF-κB can regulate apoptosis, pyroptosis and ferroptosis. GSDMD in pyroptosis and necrosome in necroptosis can cooperate to amplify inflammatory signals.

During sepsis, the cell death pathway involves complex interactions of myocardial cell death signals (Hsieh et al., 2009; Leng et al., 2021). In the development of sepsis induced myocardial injury, the activation of multiple cell death pathways may occur together and interact at various stages, and crosstalk between signal cascades is observed in the myocardial cell death pathway (Figure 8).

There is an interaction between pyroptosis and apoptosis mediated by caspases, which serve as intermediate bridges between the two processes. Caspase-1/4/5/11 mediate pyroptosis, while caspase-2/3/6/7/8/9/10 mediate apoptosis. However, it should be noted that the pathways involving the caspase family do not have strict boundaries (Galluzzi et al., 2018). Interactions between pyroptosis and apoptosis may occur in SIMI due to the presence of co-regulators such as ROS and caspases. Pyroptosis can facilitate apoptosis, leading to enhanced inflammation and organ dysfunction, potentially involving caspases and inflammatory factors. In a study by Li et al. (2019b) silencing of the interferon gene-stimulating factor (STING) exerted a significant anti-apoptotic effect in neonatal rat cardiomyocytes (NRCMs) treated with LPS. However, upregulation of NLRP3 counteracted the antiapoptotic effect of STING silencing, suggesting that activation of NLRP3 can trigger apoptosis in LPS-treated cardiomyocytes. The Maf1 protein was found to suppress the NF-κB/NLRP3 inflammatory pathway by directly binding to the promoter region of NLRP3, leading to the attenuation of apoptosis and inflammation. Furthermore, overexpression of NLRP3 reversed the protective effect of Maf1 against apoptosis and pyroptosis. These findings suggest that NLRP3 is involved in facilitating apoptosis in sepsis (Chen et al., 2020a). Recent studies have investigated the regulation of pyroptosis by apoptosis and have found that pannexin-1 accelerates NLRP3 activation during apoptosis (Chen et al., 2020b). Furthermore, cytoplasmic cytochrome C released during apoptosis has been shown to negatively regulate NLRP3 (Shi and Kehrl, 2016). However, the specific mechanisms by which apoptosis regulates pyroptosis in the context of SIMI still require further investigation.

Likewise, the interplay between pyroptosis and autophagy may play a crucial role in the pathogenesis of sepsis and its associated organ damage. Autophagy may be involved in regulating the activation of NLRP3 inflammasomes. In fact, autophagy can suppress NLRP3 inflammasome activation by removing endogenous NLRP3 inflammasome activators such as reactive oxygen species (ROS) and damage-associated molecular patterns (DAMPs), as well as NLRP3 inflammasome components (Biasizzo and Kopitar-Jerala, 2020). Moreover, NLRP3 inflammasome-mediated pyroptosis can also impact autophagy during sepsis development. In a septic cardiomyopathy model, NLRP3 knockout mice exhibited significantly lower levels of the key autophagy protein Atg7 after 24 h of CLP modeling compared to wild-type mice, indicating an inhibition of autophagy (Jin et al., 2017). These findings suggest that the interplay between pyroptosis and autophagy may be bidirectional, highlighting the complexity of the underlying mechanisms involved in sepsis pathogenesis.

Furthermore, pyroptosis and necroptosis have been shown to act synergistically in the context of SIMI. Previous studies have demonstrated that that the classical pathway of necroptosis, RIPK1/RIPK3 pathway, can collaborate with the key protein of pyroptosis, GSDMD, to amplify inflammatory signals and exacerbate tissue damage during sepsis (Chen et al., 2022b). Additionally, GPX4 has been shown to regulate both pyroptosis and ferroptosis in infection-induced multiple organ damage in sepsis, indicating that the mechanisms of pyroptosis and ferroptosis may also intersect (Zhu et al., 2019).

Interestingly, both apoptosis and necroptosis have been found to be associated with cellular damage and the activation of inflammatory cytokines, with apoptosis potentially preceding necroptosis (Speir and Lawlor, 2021). When sepsis and target organ damage occur, if cells undergo apoptosis but are not promptly phagocytosed, it may trigger the phosphorylation of RIPK and initiate necroptosis. This process ultimately leads to the rupture of the cell membrane and the release of intracellular factors (Humphries et al., 2015; Nagata, 2018). Therefore, when the mechanisms of cell death cannot be explained solely by apoptosis, it is noteworthy to consider the involvement of necroptosis (Feoktistova et al., 2021; Ofengeim and Yuan, 2013). There is evidence of cross-regulation between apoptosis and autophagy. NF-κB-activated autophagy has been shown to be closely correlated with apoptosis (Vanden Berghe et al., 2015). Beclin-1, a critical protein in both autophagy and apoptosis, serves critical function in SIMI (Lu et al., 2020). Interestingly, studies have demonstrated that suppressing Beclin-1 expression or inhibiting its activity in an LPS-induced myocardial injury model leads to the suppression of ferroptosis and impaired myocardial remodeling and systolic function (Kang et al., 2018; Yin et al., 2020). This suggests a potential intersection between apoptosis, autophagy, and ferroptosis. Furthermore, necroptosis, autophagy, and ferroptosis have been implicated in sepsis and the development of target organ dysfunction through signal transduction pathways and various proteins (Fang et al., 2020; Ma et al., 2020).

In summary, apoptosis, necroptosis, pyroptosis, autophagy, and ferroptosis do not occur in isolation but rather involve complex interactions within signaling pathways during SIMI. These multiple cell death pathways interact with each other and co-regulate inflammatory responses, oxidative stress, and other processes. However, the specific mechanisms underlying these interactions have not been fully explored. Further in-depth investigations are needed to understand the development of SIMI and the crosstalk between signaling cascades, in order to identify potential target treatments for SIMI.

## 8 Therapeutic strategies based on PCD in SIMI

Previously, we have described the pathways, related proteins, and common signaling pathways of PCD in SIMI. These various PCD pathways are intertwined with each other. Some current therapeutic strategies for SIMI are based on the understanding of these five pathophysiological mechanisms (Figure 1). The table illustrates how certain drugs can ameliorate septic myocardial injury through various mechanisms of action (Table 1). For instance, melatonin has been shown to ameliorate SIMI by activating the Nrf2-related pathway and inhibiting the formation of NLRP3 inflammasomes (Rahim et al., 2021). Additionally, melatonin can be implicated in the activation of AMPK-mediated autophagy, thereby alleviating SIMI (Di et al., 2020). Furthermore, picloram has been shown to synergize its protective effects against SIMI by weakening ferroptosis through upregulating GPX4 expression and inhibiting apoptosis through activating the PI3K/AKT signaling pathway (Xiao et al., 2023). Given the complex interplay of various types of programmed cell death in sepsis and septic myocardial injury, it is evident that a multi-targeted therapy approach for SIMI may become a prominent topic in the future. Instead of targeting a single pathway or mechanism, this approach aims to simultaneously modulate multiple targets involved in different types of programmed cell death pathways. By employing a multi-targeted therapy strategy, it becomes possible to address the intricate network of signaling cascades and molecular interactions contributing to SIMI. However, it is noteworthy to acknowledge that the development of multi-targeted therapies for SIMI requires extensive research to identify suitable targets, assess their interactions and effects, and determine the optimal combination of interventions. Furthermore, thorough preclinical and clinical studies are necessary to evaluate the safety and efficacy of such approaches before they can be considered for widespread clinical application.

**TABLE 1:** Therapeutic strategies based on PCD in SIMI.

Mechanisms	Therapy	Target/Signaling pathways	Effect for Target/Signaling pathways	*In vivo* model	*In vivo* subject	*In vitro* model	*In vitro* subject	Effect	Reference
Apoptosis	Dehydrocorydaline	TRAF6/NF-κB	Downregulation	LPS	Mouse	LPS	H9C2	Inhibition	Li et al. (2021b)
	Hesperetin	JNK/Bax	Downregulation	LPS	-	LPS	H9C2	Inhibition	Yang et al. (2014b)
	Oleuropein	NF-κB	Downregulation	CLP	Mouse	-	-	Inhibition	Xing et al. (2021)
	H2S melation	Bax caspase	Downregulation	CLP	Rat	-	-	Inhibition	Liu et al. (2019b)
	Tannic acid (TA)	ROS	Downregulation	LPS	-	LPS	H9C2	Inhibition	Yang et al. (2021)
	Oxymatrine (OMT)	TNFα/P38-MAPK/caspase	Downregulation	CLP	Rat	-	--	Inhibition	Zhang et al. (2016b)
	Shenfu (SF)	Bcl-2 caspase9	Upregulation	LPS	Mouse	-	-	Inhibition	Xu et al. (2020a)
	Xuefu Zhuyu Decoction	Caspase3	Downregulation	LPS	Rat	-	-	Inhibition	Meng et al. (2018)
	resveratrol	PI3K/AKT/mTOR	Upregulation	LPS	Rat	-	-	Inhibition	Shang et al. (2019a)
	Matrine	PI3K/AKT	Upregulation	LPS	Mouse	-	-	Inhibition	Xiao et al. (2023)
	Astaxanthin (ATX)	MAPK	Downregulation	LPS	Mouse	-	-	Inhibition	Xie et al. (2020)
	Vitamin B6	Bcl-2	Upregulation	LPS	Mouse	LPS	H9C2	Inhibition	Shan et al. (2021)
Necropotosis	PPAR-γ	RIP1/RIP3/MLKL	Downregulation	CLP	Rat	-	-	Inhibition	Peng et al. (2017)
Pyroptosis	Necrosulfonamide	GSDMD	Downregulation	LPS	Mouse	-	-	Inhibition	Rathkey et al. (2018)
	Emodin	NLRP3, GSDMD	Downregulation	LPS	Mouse	-	-	Inhibition	Dai et al. (2021)
	Vitamin C	ROS-AKT/mTOR	Downregulation	LPS	-	LPS	H9C2	Inhibition	Zhang et al. (2022)
	Irisin	NLRP3	Downregulation	LPS	Mouse	-	-	Inhibition	Li et al., 2021a- Wang et al., 2022b
	HSP70	NLRP3, caspase-1, GSDMD	Downregulation	CLP	Mouse	LPS	H9C2	Inhibition	Song et al. (2022)
	Syringaresinol (SYR)	ER/SIRT1/NLRP3/GSDMD	Downregulation	CLP	Mouse	-	-	Inhibition	Wei et al. (2021)
	Melatonin (MT)	NLRP3, caspase-1, GSDMD	Downregulation	LPS	-	LPS	H9C2	Inhibition	Rahim et al. (2021)
	Dehydrogenase (ALDH2)	NLRP3, caspase-1, GSDMD	Downregulation	LPS	Mouse	-	-	Inhibition	Zhang et al. (2023)
	Geniposide (GE)	NLRP3	Downregulation	LPS	Mouse	-	-	Inhibition	Song et al. (2020)
	Jujuboside A (JuA)	NLRP3	Downregulation	LPS	Mouse	-	-	Inhibition	Wang et al. (2023b)
	MCC950	NLRP3/Caspase-1/IL-1β	Downregulation	CLP	Rat	LPS	H9C2	Inhibition	Li et al. (2021d)
	Xinyang Tablet (XYT)	MLK3	Downregulation	LPS	Mouse	-		Inhibition	Wang et al. (2021b)
	Recombinant human Angiotensin-converting Enzyme 2 (rhACE2)	NLRP3	Downregulation	LPS	Mouse	-	-	Inhibition	Wu et al. (2023)
	Sodium tanshinone IIA Sulfonate (STS)	NLRP3	Downregulation	LPS	Mouse	-	-	Inhibition	Chen et al. (2021)
	Carvacrol (CVL)	NLRP3, caspase-1, GSDMD	Downregulation	LPS	Mouse	LPS	H9C2	Inhibition	Joshi et al. (2023)
Ferroptosis	Ferrostatin-1	TLR4/NF-κB	Downregulation	LPS	Mouse	-	-	Inhibition	Xiao et al. (2021)
	MRI-visible melanin Nanoparticles (MMPP)	GPX4	Upregulation	LPS	Mouse	-	-	Inhibition	Liu et al. (2023)
	NaHS	GPX4, SLC7A11	Upregulation	LPS	Mouse	-	-	Inhibition	Cao et al. (2022)
	Platelet-rich plasma (PRP)	SOD, GSH	Upregulation	LPS	Mouse	-	-	Inhibition	Jiao et al. (2022)
	Tectorigenin	GPX4	Upregulation	LPS	Mouse	-	-	Inhibition	Fang et al. (2023)
	Matrine	GPX4	Upregulation	LPS	Mouse	-	-	Inhibition	Xiao et al. (2023)
	Vitamin B6	Nrf2	Upregulation	LPS	Mouse	LPS	H9C2	Inhibition	Shan et al. (2021)
	Cyclovirobuxine D (CVB-D)	SLC7A11, GPX4	Upregulation	CLP	Rat	LPS	H9C2	Inhibition	Wang et al. (2023a)
Autophagy	UCP2	AMPK	Upregulation	CLP	Mouse	LPS	H9C2	Activation	
	Narciclasine	JNK	Upregulation	LPS	Mouse	-	-	Activation	Tang et al. (2021)
	Melatonic	AMPK	Upregulation	LPS	Mouse	-	-	Activation	Di et al. (2020)
	Jujuboside A (JuA)	PI3K/AKT	Upregulation	LPS	Mouse	-	-	Activation	Wang et al. (2023b)
	Sodium tanshinone IIA Sulfonate (STS)	AMPK	Upregulation	LPS	Mouse	-	-	Activation	Chen et al. (2021)
	Luteolin	AMPK	Upregulation	LPS	Mouse	-	-	Activation	Wu et al. (2020)
	Eupafolin	PI3K/AKT/mTOR	Upregulation	LPS	-	LPS	H9C2	Activation	Gao et al. (2019)
	Dexmedetomidine	PI3K/AKT	Upregulation	CLP	Rat	LPS	H9C2	Activation	Yu et al. (2019)
	Capsaicin	AMPK	Upregulation	LPS	rat	-	-	Activation	Qiao et al. (2021)

## 9 Conclusion

In the past 20 years, extensive research has been conducted to understand the mechanisms of SIMI in various fields, including proteomics and genomics. PCD has been recognized as playing a crucial role in the occurrence and progression of SIMI. The current review focuses on discussing the mechanisms of PCD in SIMI, the interaction among different PCD pathways, the novel therapeutic strategies being explored, and future development prospects. However, the specific mechanism of PCD in cardiomyocytes during SIMI remains poorly understood. For instance, it is unclear where these five modes of PCD occur and whether they are equally involved in SIMI. Furthermore, there is still no identified specific and comprehensive signaling pathway that fully explains the occurrence and development of SIMI. Many signaling molecules necessary for the pathway’s understanding are still unidentified despite the progress made so far. Hence, it is essential to prioritize and further clarify the specific molecular mechanisms of PCD in SIMI to formulate more targeted therapeutic strategies for SIMI in the future.

## Figures and Tables

**Table:** 

**ICUs**	intensive care units
**MODS**	multiple organ dysfunction syndrome
**SIMI**	sepsis-induced myocardial injury (SIMI)
**DAMPs**	damage-associated molecular patterns
**PCD**	programmed cell death
**TRADD**	tumor necrosis factor receptor-associated death domain
**SMAC**	second mitochondria-derived activator of caspase
**Apaf-1**	apoptotic protease activating factor-1
**FADD**	FAS-associated death domain
**LPS**	lipopolysaccharide
**TNF**	tumor necrosis factor
**DUSP1**	dual specificity phosphatase-1
**NF**	nuclear factor
**NLS**	nuclear localization sequence
**TLR**	toll like receptor
**RIPK**	receptor interacting protein
**IAPs**	inhibitor of apoptosis proteins
**CYLD**	cylindromatosis
**MLKL**	mixed lineage kinase domain-like
**SOFA**	sequential organ failure assessment
**Nec-1**	necrostatin-1
**CLP**	cecal ligation and puncture
**GSMD**	gasdermin
**NLRP3**	NOD-like receptor protein 3
**NLRC4**	nucleotide-binding oligomerization domain, leucine-rich repeat and caspase recruitment domain-containing 4
**AIM2**	absent in melanoma 2
**ASC**	apoptosis-associated speck-like protein containing CARD
**BMECs**	brain microvascular endothelial cells
**Nrf2**	nuclear factor erythroid 2-related factor
**CTRP1**	C1q/tumor necrosis factor-related protein 1
**GPX4**	glutathione peroxidase 4
**CoQ**	coenzyme Q
**MVA**	mevalonate
**FSP1**	ferroptosis suppressor protein 1
**NADPH**	nicotinamide adenine dinucleotide phosphate hydrogen
**IPP**	isopentenyl pyrophosphate
**PTGS2**	prostaglandin endoperoxide synthase 2
**MDA**	malondialdehyde
**DXZ**	dexrazoxane
**SLC7A11**	solute carrier family 7 member 11
**SLC3A2**	solute carrier family 3 member 2
**GSH**	glutathione
**HO-1**	heme oxygenase-1
**SIRT**	Sirtuin
**NETs**	neutrophil extracellular traps
